# Pathway Dependence in Redox‐Driven Metal–Organic Gels

**DOI:** 10.1002/chem.202001051

**Published:** 2020-04-30

**Authors:** Santanu Panja, Dave J. Adams

**Affiliations:** ^1^ School of Chemistry University of Glasgow Glasgow G12 8QQ UK

**Keywords:** kinetic control, metal–organic gels, pathway dependence, redox responsiveness, supramolecular gels, swelling

## Abstract

Pathway dependence is common in self‐assembly. Herein, the importance of pathway dependence for redox‐driven gels is shown by constructing a Fe^II^/Fe^III^ redox‐based metal–organic gel system is shown. In situ oxidation of the Fe^II^ ions at different rates results in conversion of a Fe^II^ gel into a Fe^III^ organic gel, which controls the material properties, such as gel stiffness, gel strength, and an unusual swelling behaviour, is described. The rate of formation of Fe^III^ ions determines the extent of intermolecular interactions and so whether gelation or precipitation occurs.

## Graphical abstract

**Kinetic control**: Pathway dependence is common in self‐assembly. Here, the importance of pathway dependence for redox‐driven gels is discussed by constructing a Fe^II^/Fe^III^ redox‐based metal–organic gel system.

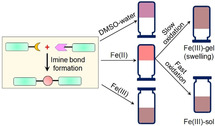

Supramolecular low molecular weight hydrogels (LMWGs) formed by the self‐assembly of small organic molecules induced by non‐covalent interactions are fascinating smart materials, which have multifunctional applications.^1^ Of the different kinds of supramolecular gels, metal–organic gels have received significant interest in recent years because of their widespread applications, particularly in optoelectronics, pharmaceuticals and catalysis.^2^ Metal–organic gels are a special class of supramolecular gels that incorporate a metallic element into the ligand during self‐assembly. Conceptually, metal–organic gels are synthesized based on strong metal–ligand interactions, in which the organic ligand may compose of a single component or can be derived from the reaction between multiple functional groups. Incorporation of metal ions into the organic frameworks often dramatically changes the optical and chemical properties of the ligand and therefore can be used as a powerful strategy to modify the material properties.2a, 2b, 3

One interesting property of supramolecular gels is their responsiveness towards various stimuli including heat, pH, irradiation, chemical entities, and redox reactions.3b, 4 Redox responses make the gel systems desirable for biomimicry, as well as for numerous possible applications.4f, 5 However, most of the redox‐fuelled gels found in the literature are polymeric in nature and are usually developed from the intermolecular disulfide exchange reaction‐based molecular systems.4f, 5c, 5d, 5e, 5g Therefore, designing and construction of new supramolecular low molecular weight redox‐based gels is highly desirable; these are expected to have very different underlying properties.^6^

A key issue for many supramolecular gels is that the properties significantly depend on the preparative pathway.^7^ Because of such effect, even though the composition of the final materials remains same, the material properties can vary depending on the self‐assembly kinetics.7a Gels formed at a high rate are often kinetically trapped, which means that they can be hard to reproduce and control. To avoid this kinetic trapping during the gelation process, the environmental conditions need to be well controlled to achieve homogeneous and reproducible gels.

Herein, we designed a new redox‐responsive metal–organic hydrogel system and discuss the pathway dependence of these redox‐based gels (Figure 1). Unlike other redox systems, instead of using sulfide/disulfide‐based ligands,4f, 5c, 5d, 5g herein, we utilize dynamic imine bond formation between an aldehyde (**1**) and an amine (**2**) as the key chemical reaction to synthesize the ligand (**3**). To make the organic framework redox responsive, we incorporated Fe^II^ ions into the gel medium. In situ oxidation of the Fe^II^ ions by an oxidising agent results in formation of a Fe^III^–organic gel. The final properties of the Fe^III^ gel significantly depend upon the rate of oxidation of Fe^II^. Although a slow rate of oxidation gives Fe^III^ gels with high stiffness, a very fast oxidation drives the system towards precipitation. Precipitation also occurred on direct treatment of the mixture of the aldehyde (**1**) and amine (**2**) with Fe^III^. Hence, we showed that we can prepare Fe^III^ metallogels, which cannot be prepared directly by controlling the reaction pathway. In some cases, we also find that the materials exhibit a highly unusual swelling, which is very uncommon for such supramolecular gels.

**Figure 1:**
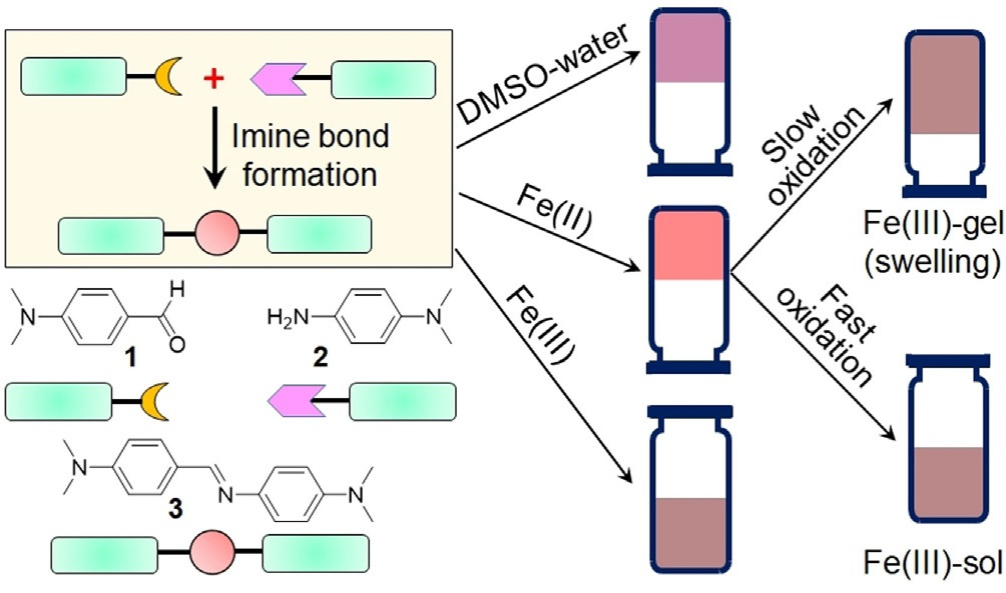
Cartoon representing the phase transformations of the mixture of **1** and **2** under different conditions (the pictures of the inverted vials represent gel states).

To prepare the gel, we employed dynamic imine bond formation^8^ reaction between 4‐(dimethylamino)benzaldehyde (**1**) and *N*,*N*‐dimethyl‐*p*‐phenylenediamine (**2**) in DMSO/H_2_O (25:75 v/v). When a mixture of equimolar amount of **1** and **2** (0.134 m) in DMSO is diluted with water, a brown self‐supporting gel was rapidly formed (Figure 2 a). The gelation process was followed by rheology. By time sweep rheology, initially the storage modulus (G′) was considerably higher than the loss modulus (G′′), indicating that gelation was very quick and occurred before the measurement could be begun (Figure 2 a). The gel continued to evolve with time and G′ and G′′ reach a plateau after approximately 14 hours (Figures 2 b and S1 in the Supporting Information). The gel exhibits a high stiffness (≈2×10^5^ Pa), but starts to collapse at a low strain of approximately 0.2 % (the critical strain; Figures 2 c and S1).

**Figure 2:**
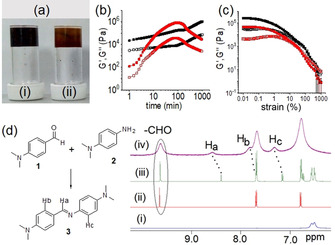
(a) Photograph of the hydrogels obtained from the mixture of **1** and **2** in absence (i) and presence (ii) of Fe^II^. (b) Variation of G′ (closed symbol) and G′′ (open symbol) with time for the mixture of **1** and **2** in absence (black) and presence (red) of Fe^II^. (c) Strain sweeps of the gels prepared in absence (black) and presence (red) of Fe^II^ after 16 hours. The closed symbols represent G′, open symbols G′′. (d) Partial ^1^H NMR (in [D_6_]DMSO) spectra of (i) **2**; (ii) **1**; (iii) gels obtained from the mixture of **1** and **2** in absence of Fe^II^; and (iv) gels obtained from the mixture of **1** and **2** in presence of Fe^II^. In all cases (a–d), the initial concentrations of **1**, **2**, and Fe^II^ are 0.134 m.

To characterize the chemical component responsible for gelation, ^1^H NMR spectroscopy and high‐resolution mass spectroscopy (HRMS) of the gel state were performed (Figure 2 d). By ^1^H NMR spectroscopy, the appearance of a new peak at 8.39 ppm clearly demonstrated the imine bond formation between the aldehyde and amine. Integration of the ^1^H NMR spectra showed around 26 % conversion to the imine **3** after 16 hours. This presumably represents the position of the equilibrium of the reaction under these conditions, because imines are susceptible to hydrolysed in water.^8^, ^9^ The imine bond formation was further confirmed by recording the HRMS of the gel (Figure S2 in the Supporting Information). The appearance of the mass at 268.1792 shows the formation of compound **3** [expected mass=268.1814 for the formula (*M*+H)^+^] in the gel state. Moreover, by FTIR spectroscopy, the stretching signal for the aldehyde carbonyl of **1** appeared at 1656 cm^−1^, whilst in the gel state it remained almost unaffected (Figure S3 in the Supporting Information). However, a broad peak appeared at 1680 cm^−1^ for the C=N bond, again confirming the formation of the imine **3** in the gel state.

We incorporated Fe^II^ ions (as sulfate salt) into the gel medium to convert this supramolecular gel into a redox responsive metal–organic gel.2c, 5a, 5f, 10 We used 0.134 m of Fe^II^ to prepare the Fe^II^ gel (1  molar equivalent with respect to the aldehyde). Addition of aqueous solution of Fe^II^ to the mixture of **1** and **2** not only modified the gelation kinetics, but also changed the final mechanical properties of the gels. Time sweep rheology indicated that the initial values of both G′ and G′′ were significantly lower compared to the case when Fe^II^ was absent (Figures 2 b and S1). With time, both G′ and G′′ stated to increase rapidly. After approximately 2 hours, G′ and G′′ started to decrease and became almost constant after approximately 13 hours (Figure S1 in the Supporting Information). The presence of Fe^II^ resulted in around approximately six times decrease in both G′ and G′′ of the gel (Figures 2 c and S1). However, no significant change in gel strength (critical strain) was observed (Figure 2 c). Surprisingly, when we tried to make a control gel with preformed imine **3**, no gel formation was noticed either in absence or presence of Fe^II^ (Figure S4 in the Supporting Information). Compound **3** is poorly soluble in DMSO. Upon addition of H_2_O to a suspension of **3** in DMSO, a yellow and orange yellow precipitate was formed in absence and presence of Fe^II^ respectively. Hence, in situ formation of **3** is necessary for gelation to occur.

The presence of Fe^II^ also changed the visual appearance of the gel (Figure 2 a). The colour of the gel changed from brown to reddish brown in presence of Fe^II^. UV/Vis and emission spectroscopy measurements of **1** and **2** were conducted under different conditions to highlight the aggregation properties (Figures S5 and S6 in the Supporting Information). By UV/Vis analysis, **1** and **2** exhibited absorption at 353 and 305 nm, respectively. In comparison, the gel state obtained from the mixture of **1** and **2** showed a strong absorption at 338 nm with two shoulder peaks at 310 and 434 nm. Time‐dependent emission experiments showed that as the reaction proceeds, the strong emission of the aldehyde at 410 nm started to decrease, and a new band appeared at 468 nm. In presence of Fe^II^, the absorption peak at 310 nm became more intense, whereas the shouldering at 434 nm remained unaffected. By fluorescence, the emission of the gel at 468 nm blueshifted by 8 nm in presence of Fe^II^ along with the generation of a new band at 550 nm. These data suggest existence of different intermolecular interactions in the gel matrices formed in absence and presence of Fe^II^. To confirm this, the ^1^H NMR spectrum of the Fe^II^ gel was superimposed with that obtained in absence of Fe^II^ (Figure 2 d). Comparison of the data shows that the signal for the imine proton H_a_ of **3** moved to the downfield region by 0.2 ppm due to the interaction with Fe^II^. Moreover, due to metal coordination, the aromatic protons H_b‐c_ also showed approximately 0.2 ppm downfield shift. Interestingly, while the signals for aromatic protons, as well as the carbonyl −CH of **1**, exhibited no shift in ^1^H NMR upon interaction with Fe^II^, the aromatic protons of **2** became broad and shifted downfield by 0.2 ppm. These results indicate that Fe^II^ not only binds with the imine bond of **3**, but also interacts with the amine functionality of **2**. By HRMS, no evidence of formation of **3**–Fe^II^ complex was found (Figure S7 in the Supporting Information). This indicates that although the interaction of the imine bond with Fe^II^ ion is subtle, it causes significant change at the macroscopic level.

The presence of Fe^II^ ions makes the gel medium redox responsive.2c, 5a, 5f, 10 Practical uses of Fe^II^/Fe^III^ redox systems involving LMWGs are limited in the literature. For example, recently, Das et al. reported a reusable transient hydrogel system based on Fe^II^/Fe^III^ redox conversion and explored those transient aggregates in mimicking peroxidase activity.5a Panja and Ghosh utilized a Fe^II^ metallogel for visual recognition of H_2_O_2_ from other reactive oxygen species (ROS) by performing Fenton reaction inside the gel medium.^11^ Inspired by their work, we attempted to convert our Fe^II^ gel into a Fe^III^ gel through an in situ oxidation of the Fe^II^ ions by different oxidizing agents.

Prior to this, we investigated the role of dissolved oxygen on our Fe^II^ gel. For this purpose, instead of deionized water, we used degassed, deionized water to prepare the gel. Rheological studies showed that the rheological moduli, as well as viscosity, follow similar trends as in the case with normal water. Interestingly, final values of both G′ and G′′ of the gel formed with normal water are considerably lower than the gel formed with degassed water (Figure S8 in the Supporting Information). However, no significant change in the gel strength (the critical strain) was observed. These results point out that the dissolved oxygen has a subtle effect on the stability of Fe^II^ ions and presumably oxidise some Fe^II^ ions into Fe^III^ ions inside the gel medium, resulting in the decrease in stiffness of the material (by ≈3 times). However, no significant change in the absorption and emission spectra of the gels were noticed (Figure S9).

Next, we used NaNO_2_ (0.067 m) as an in situ oxidizing agent and monitored the self‐assembly kinetics by time sweep rheology. Because the NaNO_2_ is a mild oxidant, it causes slow conversion of Fe^II^ ions into Fe^III^. By time sweep rheology, at the early stages, a slight increase in the rate of increase of both G′ and G′′ was noticed (Figure 3 a). Interestingly, after reaching the maxima, the rheological moduli started to decrease earlier than the case with no oxidizing agent before the values become almost constant after approximately 12 hours. Viscosity data recorded over time follows a similar trend as that of rheology (Figure S10 in the Supporting Information). Instead of NaNO_2_, when same concentration of H_2_O_2_ (0.067 m) was used, which is a stronger oxidant, the self‐assembly kinetics behave differently (Figures 3 b and S10 in the Supporting Information). In this case, the variation of the rheological moduli was straightforward, in which G′ and G′′ increase as the aggregation proceeds and finally reached the plateau after approximately three hours. However, in both cases, formation of Fe^III^ resulted in a significant decrease in the stiffness of the final gels, whereas the extent of reduction in the values of G′ depends on the rate of oxidation of Fe^II^ (Figures 3 c, S11, S12, and Table S1 in the Supporting Information). While a slow oxidation of Fe^II^ by NaNO_2_ causes approximately four times reduction in G′, fast oxidation involving H_2_O_2_ resulted in a tenfold decrease in the stiffness of the gel compared to the pristine Fe^II^ gel. However, irrespective of rate of oxidation of Fe^II^, the final Fe^III^ gels showed approximately four times increase in strength of the materials.

**Figure 3:**
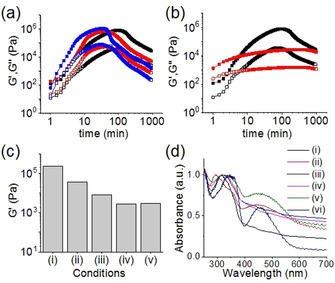
Variation of G′ (closed symbol) and G′′ (open symbol) with time for the mixture of **1**, **2** and Fe^II^ in presence of redox reaction involving (a) NaNO_2_ and (b) H_2_O_2_. The black data is for no oxidising agent, the red data for 0.067 m and the blue data for 0.134 m oxidising agent. (c) Bar graph representing the stiffness (G′) of the final gels obtained from: (i) the mixture of **1** and **2**; (ii) the mixture of **1**, **2** and Fe^II^; (iii)–(v) the mixture of **1**, **2** and Fe^II^ in presence of redox reaction involving 0.067 m of NaNO_2_ (iii); 0.067 m of H_2_O_2_ (iv) and 0.134 m of NaNO_2_ (v). (d) Normalized UV/Vis spectra of the gel (i) and sol (ii) obtained from the mixture of **1** and **2** in presence of Fe^II^ and Fe^III^ respectively. (iii–v) Normalized UV/Vis spectra of the gels obtained from the mixture of **1**, **2** and Fe^II^ in presence of redox reaction involving 0.067 m of NaNO_2_ (iii), 0.134 m of NaNO_2_ (iv) and 0.067 m of H_2_O_2_ (v). (vi) Normalized UV/Vis spectra of the sol obtained from the mixture of **1**, **2** and Fe^II^ in presence of redox reaction involving 0.134 m of H_2_O_2_. For (a)–(d), initial concentrations of **1**, **2** and Fe^II^ are 0.134 m.

We further increased the rate of oxidation of Fe^II^ by increasing the concentration of the oxidizing agents. Similar trends in G′, G′′, and viscosity were monitored when we increased the initial concentration of NaNO_2_ from 0.067 m to 0.134 m (Figures 3 a and S10 in the Supporting Information). The final values of G′ and G′′ of the gels concomitantly decreases with an increase in the initial concentration of NaNO_2_ (Figures 3 c, S11, and Table S1 in the Supporting Information). Notably, when we increased the concentration of H_2_O_2_ (0.134 m), instead of a gel, precipitation occurred (Figure S12). These results suggest that the formation of Fe^III^ gel depends significantly on the rate of oxidation of Fe^II^. Interestingly, direct treatment of the mixture of **1** and **2** with Fe^III^ (0.134 m) produced precipitation (Figure S12). Correlation of these results indicates a complex mechanism for the formation of Fe^III^ gels via oxidation processes, in which two phenomena are occurring simultaneously by the formation of the imine **3** and the conversion of Fe^II^ to Fe^III^. A slow conversion to Fe^III^ allows formation of continuous network structures involving **3**, whereas fast oxidation drives the system towards kinetically trapped states, in which the intermolecular interactions involving Fe^III^ were strong enough to produce precipitation.^12^

The visual appearance of the gels also depends on the initial reaction conditions. Oxidation of Fe^II^ either by NaNO_2_ or H_2_O_2_ turned the reddish brown Fe^II^ gels into deep brown Fe^III^ gels (Figure S12 in the Supporting Information). However, these gels behaved differently by spectroscopy. In fluorescence, the emission of all Fe^III^ gels was quenched at 550 nm (Figure S13). In UV/Vis data, all the Fe^III^ gels showed absorption in the region 330–360 nm (Figure 3 d). Interestingly, the absorption intensity in this region increased as the formation of Fe^III^ was faster. When the rate of oxidation of Fe^II^ was significantly high, particularly with H_2_O_2_, a distinct peak at 550 nm appeared. A similar spectral appearance was also observed for the sol obtained from direct treatment of Fe^III^ with **1** and **2**. These results suggest that when the rate of oxidation of Fe^II^ is extremely high, almost all the Fe^II^ is converted to Fe^III^ very rapidly and the binding interactions follow the similar pattern as in the case with Fe^III^ alone. To get more insight, FTIR studies were conducted with the metallogels prepared under different conditions (Figure S14 in the Supporting Information). FTIR studies showed that irrespective of rate of oxidation, for the Fe^III^ gels (as well as sols), C=N bond formation occurs as in all cases with appearance of a broad peak at 1680 cm^−1^. To understand the interactions with Fe^III^, we attempted to collect ^1^H NMR spectra of the Fe^III^‐containing gels and sols obtained under different conditions. First, we recorded the ^1^H NMR spectrum of **1** and **2** in presence of NaNO_2_ and H_2_O_2_ separately to investigate if oxidation leads to any chemical changes in the systems. From Figures S15–S17 in the Supporting Information, it is evident that no chemical changes occur to **1** and **2** in presence of the oxidizing agents. Similarly, the presence of NaNO_2_ does not alter the composition of the gel formed from **1** and **2** (Figure S18). We were unable to record the ^1^H NMR spectra of the gels obtained from mixture of Fe^II^ with **1** and **2** in the presence of NaNO_2_. However, HRMS experiments showed formation of **3** both in absence and presence of Fe^II^ involving NaNO_2_ (Figure S19). For the systems formed from **1** and **2** involving H_2_O_2_, the aromatic protons of **2** became broad both in absence and presence of Fe^II^ (Figure S20 in the Supporting Information). A similar spectral appearance was observed in the sol obtained from direct treatment of the mixture of **1** and **2** with Fe^III^ (Figure S21). Correlation of the results from Figures S16 and S20 shows that H_2_O_2_ readily reacts with Fe^II^ (as the peak at 10.48 ppm corresponds to H_2_O_2_ disappeared in presence of Fe^II^) but causes no chemical changes to **3**. HRMS analysis also confirms the formation of compound **3** in all cases (Figures S22, S23). Furthermore, analysis of ^1^H NMR spectra showed the presence of chemical analytes (oxidizing agents, metal ions) have no significant effect on conversion of **3** (the percentage conversion of **3** varies between 20–26 % in all cases). The slight variations in conversion of **3** is probably due to the fact that during recording of the NMR spectra, a small amount of hydrolysis may occur.8b, 9 Notably, in the mixture of **1** and **2**, unlike Fe^II^, no shift of the imine proton H_a_ (Figures S20, S21) was observed whether Fe^III^ is used directly or generated in situ by oxidation of Fe^II^. Hence, formation of **3** occurred in all cases, and the interaction of **3** with Fe^III^ is not the only the determining factor for the formation a gel or sol, but instead depends upon the assembly of the underlying structures, in which the rate of formation of Fe^III^ also determines how the Fe salt interacts with the fibres. SEM images of the gels (and sols) clearly demonstrate different aggregation depending upon the preparation pathways (Figure S24 in the Supporting Information).^12^, ^13^

The resulting Fe^III^ gels showed unusual swelling behaviour depending on the rate of oxidation of Fe^II^ (Figure 4 a). The volume of the Fe^II^ gels increases on conversion to the Fe^III^ ions by NaNO_2_ and the degree of swelling is proportional to the initial concentration of NaNO_2_ (Figure 4 b and c). When 0.067 m of NaNO_2_ was used as oxidant, the resulting Fe^III^ gel showed approximately 23 % increase in volume compared to the pristine Fe^II^ gel. An increase in NaNO_2_ concentration from 0.067 to 0.134 m caused a further increase in volume of the gel (≈38 %). Figure 4 c shows the increase in gel volume with time under different rate of oxidation of Fe^II^. Interestingly, when H_2_O_2_ was used as oxidant, no such swelling was noticed.

**Figure 4:**
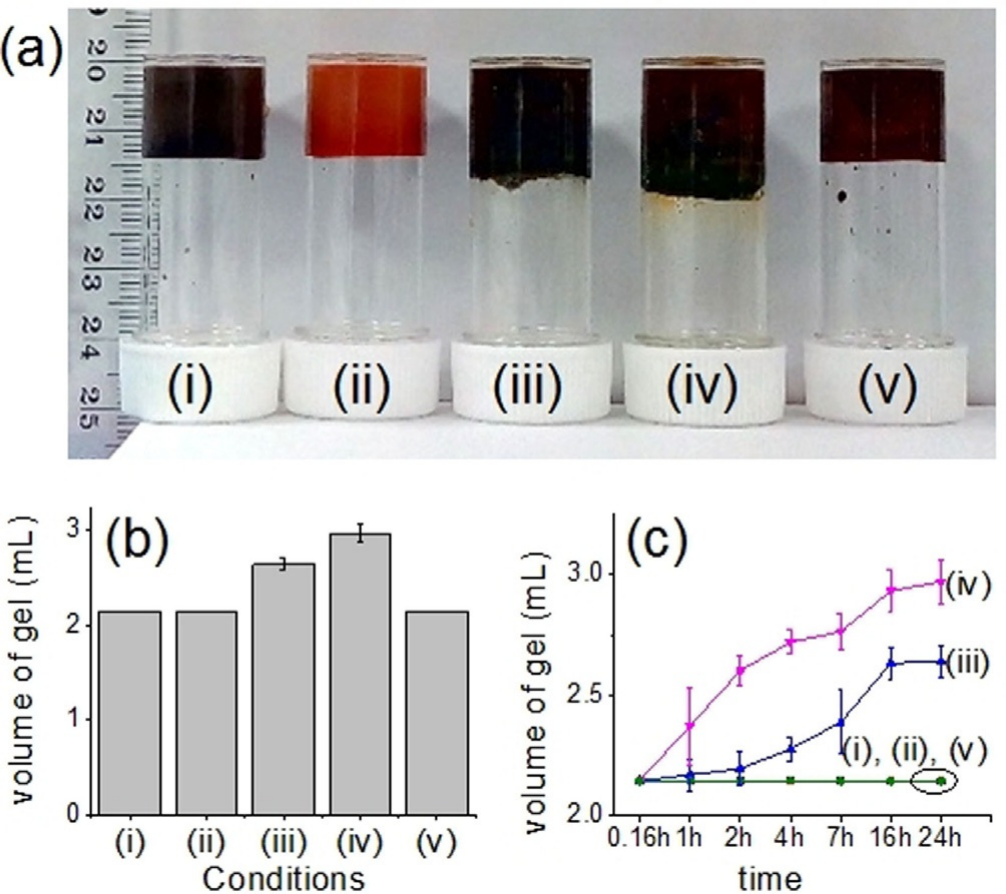
(a) Photograph representing the pathway‐driven swelling of the gels. The gels are prepared from **1** and **2** in absence (i) and presence (ii) of Fe^II^. In situ oxidation of the Fe^II^ gels by (iii) 0.067 m of NaNO_2_, (iv) 0.134 m of NaNO_2_, and (v) 0.067 m of H_2_O_2_ gives Fe^III^ gels. (b) Bar graph representing the final volume of the respective gels obtained from (a). (c) Time‐variable change in the volume of the respective gels from (a). For (a)–(d), initial concentrations of **1**, **2**, and Fe^II^ are 0.134 m.

We highlight that swelling of such a supramolecular, low molecular weight gel is very unusual. Normally, such swelling is limited to cross‐linked polymer gels. To determine the reason, polarising optical microscopic (POM) images of the gels were recorded, which showed the existence of spherical gas bubbles inside the gel medium obtained from NaNO_2_ oxidation (Figure 5). The gas bubbles are formed because of the generation of NO and NO_2_ due to the redox reaction,^14^ which create internal stresses resulting swelling.^15^ The density of the gas bubbles increases with as the increase in initial NaNO_2_ concentration, which governs the amount of volume increase in the Fe^III^ gels. In comparison, no such gas bubbles were observed in the POM images of other metallogels. The ability of the gels towards swelling before the destruction was also verified (Figure S25 in the Supporting Information). For this purpose, we increased the initial concentration of NaNO_2_ further. Swelling of the gel occurred up to a concentration of 0.134 m of NaNO_2_. Above this concentration of NaNO_2_, the volume of the gels increases, but some amount of the gel from the upper surface was destroyed and appeared as sol upon inversion of the vials. These observations suggest that the gel network is strong enough to allow swelling until a certain point, after which the internal stresses produced by the air bubbles becomes predominant and causes deformation of the network structures at the upper surface although the rest of gels remained intact.

**Figure 5:**
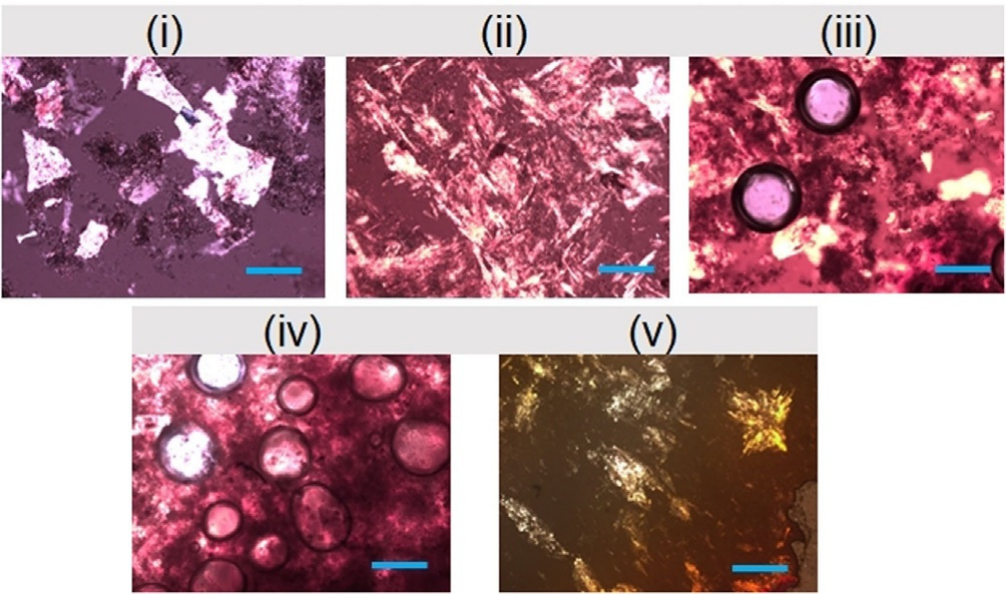
POM images of the gels (the scale bars represent 0.2 mm). The gels are prepared from **1** and **2** in absence (i) and presence (ii) of Fe^II^. In situ oxidation of the Fe^II^ gels by (iii) 0.067 m of NaNO_2_, (iv) 0.134 m of NaNO_2_ and (v) 0.067 m of H_2_O_2_ gives Fe^III^ gels. For (i)–(v), initial concentrations of **1**, **2** and Fe^II^ are 0.134 m.

In conclusion, we have shown that the pathway dependence is applicable to the redox‐driven gels by utilizing a Fe^II^/Fe^III^ redox‐based metal–organic gel system. To establish this, we utilize dynamic imine bond formation between an aldehyde (**1**) and an amine (**2**) as the key chemical reaction and incorporated Fe^II^ ions into the gel medium during the self‐assembly process. Significantly, direct preparation of the Fe^III^‐gel from the mixture of **1**, **2** and Fe^III^ ions is not feasible in our case. However, in situ oxidation of the Fe^II^ ions by various oxidising agent results in conversion to a Fe^III^‐organic gel, where the material properties like gel stiffness, gel strength, swelling etc. can be controlled just by controlling the rate of oxidation of the Fe^II^ ions. We established that the rate of formation of Fe^III^ ions actually determines the extent of intermolecular interactions whether to produce gels or precipitations. Hence, for the Fe^III^‐metallogels, which cannot be prepared directly, we can achieve those gel states in an indirect way by employing a redox reaction. We envisage that, our approach will open up opportunities to construct new functional redox gels.

## Conflict of interest

The authors declare no conflict of interest.

## Supporting information

As a service to our authors and readers, this journal provides supporting information supplied by the authors. Such materials are peer reviewed and may be re‐organized for online delivery, but are not copy‐edited or typeset. Technical support issues arising from supporting information (other than missing files) should be addressed to the authors.

Supplementary
