# Supplementary material for: Pathway Dependence in Redox‐Driven Metal–Organic Gels
Source: Chemistry. 2020 Apr 30;26(28):6130–5. doi: 10.1002/chem.202001051 (PMC7317820; doi:10.1002/chem.202001051)
Supplement: Supplementary file 1 — Supplementary [file CHEM-26-6130-s001.pdf]

Chemistry–A European Journal

Supporting Information

Pathway Dependence in Redox-Driven Metal–Organic Gels

Santanu Panja and Dave J. Adams*[a]

Table of Contents

Materials and synthesis 3

Experimental Details 5

Supplementary Figures 7

Materials: 4-(Dimethylamino)benzaldehyde (**1**) and *N*,*N*-dimethyl-p-phenylenediamine (**2**) were purchased from Sigma Aldrich and used as received. Iron(II) sulfate heptahydrate ($FeSO_4$•$7H_2O$, ACS, 99%) and Iron(III) sulfate hydrate ($Fe_2(SO_4)_3$•xH_2O, reagent grade) were obtained from Alfa Aesar. **CAUTION** - metal solutions are toxic and are handled carefully. $NaNO_2$ (99.999% trace metals basis) and H_2O_2 (30%) were obtained from Sigma Aldrich and VWR chemicals, respectively. Deionised water was used throughout all experiments. D_2O and DMSO-d_6 were purchased from Sigma Aldrich.

Syathesis of *N*[1]-(4-(dimethylamino)benzylidene)-*N*[4],*N*[4]-dimethylbenzene-1,4-diamine (3): To a stirred solution of 4-(Dimethylamino)benzaldehyde (**1**) (0.5 g, 3.35 mmol) in dry methanol (15 mL) was added a solution of *N*,*N*-dimethyl-p-phenylenediamine (**2**) (0.57 g, 4.18 mmol) in dry methanol (15 mL) at room temperature. The reaction mixture was stirred for 20 h. A precipitation appeared which was filtered and washed several times with methanol to obtain pure compound **3** in appreciable yield (0.59 g, 66%).
^1H NMR (DMSO-d_6, 400 MHz, ppm): δ 8.42 (s, 1H), 7.69 (d, 2H, J = 8 Hz), 7.16 (d, 2H, J = 12 Hz), 6.78-6.71 (m, 4H), 2.99 (s, 6H), 2.90 (s, 2H). ^{13}C NMR cannot be recorded because of poor solubility of the compound in DMSO-d_6. HRMS (ESI) m/z: $[M+H]^+$ calcd for $C_{17}H_{22}N_3$ 268.1814; found 268.1775.

^1H NMR of compound **3** in DMSO-d_6.

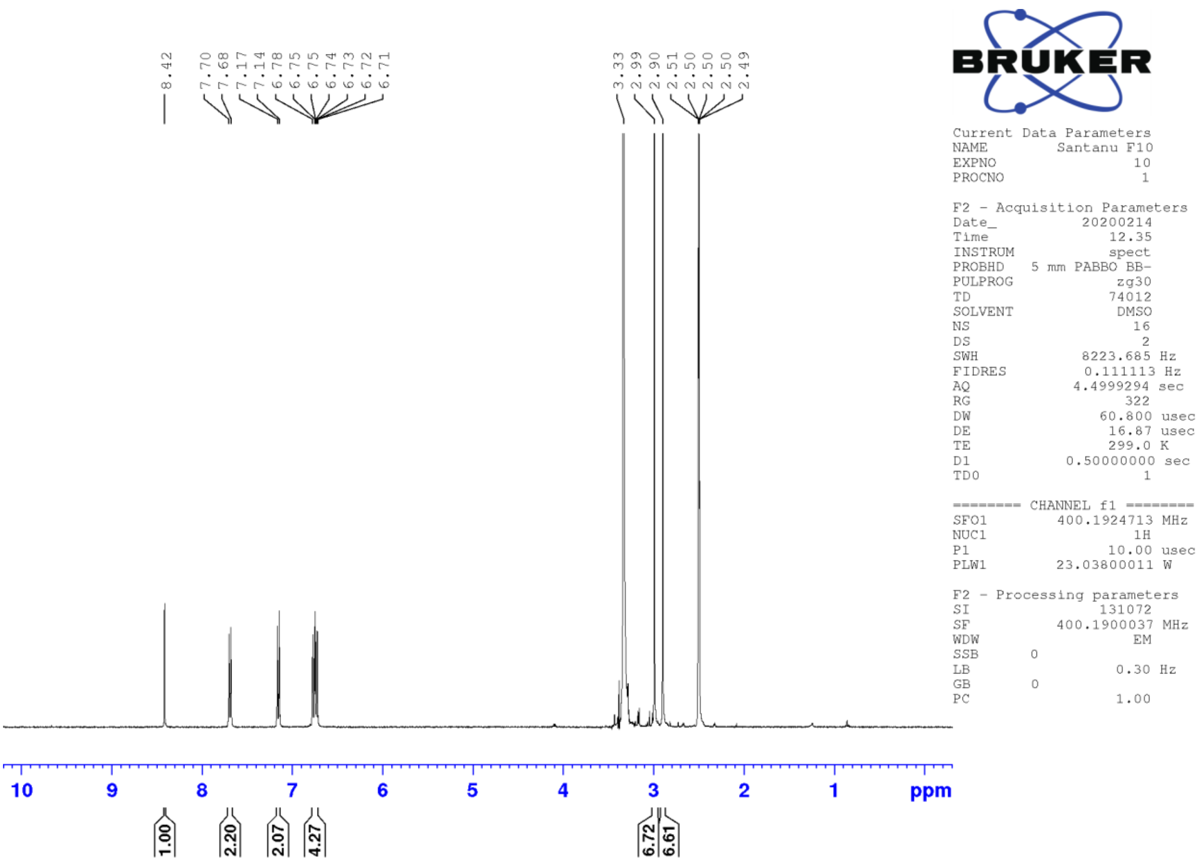

HRMS spectrum of compound **3**.

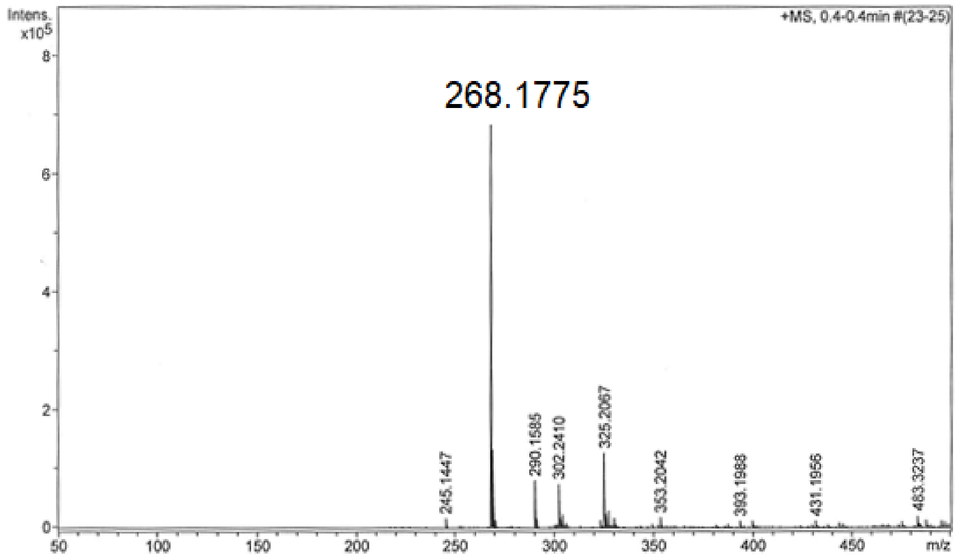

Experimental details

Preparation of Gels

Gels were prepared from an equimolar mixture of **1** and **2** (0.134 M) in DMSO/H_2O (25/75, v/v) in the presence and absence of metal ions [Fe(II) or Fe(III)] under different conditions. Stock solutions of **1** and **2** were prepared in DMSO at the concentrations of 1.34 M and 2 M respectively. Stock solutions of Fe(II) and Fe(III) were prepared at the concentrations of 0.179 M in water. To prepare the gels in absence of metal ions, 0.20 mL of solution of **1** was diluted with 0.166 mL of DMSO. To this solution, 0.134 mL of solution of **2** was added and vortexed for 10 seconds. To this mixture, 1.50 mL of of H_2O was added in one aliquot. Therefore, the ratio of DMSO and water was 25:75, and the concentrations of both **1** and **2** were 0.134 M.

To prepare the gels (or sols) in presence of Fe(II) [or Fe(III)] directly, instead of H_2O, 1.50 mL of an aqueous solution of the metal ions (0.179 M)) was added to 0.50 mL of the DMSO solution of **1** and **2**. The mixture of of **1** and **2** was prepared by following same procedure as discussed above. Therefore, in the metallogels (or sols), initial concentration of the metal ions was 0.134 M. In case of preparatiopn of Fe(II) gels in absence of dissolved oxygen, the following procedure was used. The dissolved oxgens present in water was removed by bubbling N_2 gas into the water for 3 hours fitted with a vacuum pump. This deoxygenated water was used to prepare the Fe(II) solution instead of normal deionized water.

The oxidation of the Fe(II) ions was carried out by the following procedure. Initially, the stock solutions of $NaNO_2$ and H_2O_2 were prepared at a concentrations of 2 M in water. The mixture of **1** and **2** was prepared in DMSO by following same procedure as discussed above. To this mixture, either 0.067 mL or 0.134 mL of $NaNO_2$ or H_2O_2 (as required) was added followed by imidiate addition of 1.433 mL or 1.366 mL of the Fe(II) solutions (0.187 M or 0.196 M, as required), respectively. Therefore, in all cases, the initial concentrations of **1**, **2** and Fe(II) were 0.134 M, and the initial concentrations of $NaNO_2$ or H_2O_2 were 0.067 M or 0.134 M (as required). The ratio of DMSO and H_2O was 25:75. The solutions of **1**, **2**, Fe(II), Fe(III), $NaNO_2$ and H_2O_2 were prepared freshly before each experiments.

Rheological measurements: All rheological measurements were undertaken on an Anton Paar Physica MCR 301 rheometer at 25 °C. Strain, frequency, temperature and time sweeps were performed using a vane and cup geometry. Strain sweeps were performed at 10 rad/s from 0.01 % to 1000 % strain. Frequency sweeps were carried out from 1 rad/s to 100 rad/s at 0.2 % strain. All samples were left ~16 hours before being measured. Time sweeps were performed at an angular frequency of 10 rad/s and with a strain of 0.5%. For all experiments, gels were prepared in a 7 mL Sterilin vials keeping the same volumes of the components as mentioned earlier.

Scanning Electron Microscopy (SEM): To prepare samples for SEM experiments, gels were deposited onto glass cover slips which were stuck onto aluminium SEM stubs using sticky carbon tabs and left to dry for overnight. Scanning electron microscopy images were obtained using an XL30 ESEM Phillips tungsten filament electron microscope with a secondary electron detector operating at 20 kV after gold coating (for 80 seconds) using a Polaron SC7640 sputter coater.

Polarising optical microscopy

Optical microscope images were collected under polarised light using a Nikon Eclipse LV100 microscope with a Nikon Plan ELWD 10x/0.30 lens attached to an Infinity2-1C camera. For all experiments, gels were prepared in a 7 mL Sterilin vials keeping the same volumes of the components as mentioned earlier. Then small amounts of the gels were deposited onto glass microscope slides before imaging.

NMR specroscopy

^{1}H NMR spectra were recorded on a Bruker Avance III or Avance III HD 400 or 500 MHz instruments. For ^{1}H NMR, initially gels (or sols) were prepared using DMSO-d_6 and D_2O following the same methodology as described above. Then, specific amounts of the gels (or sol) were dissolved in DMSO-d_6 in such a way that the concentration of the gels became 318 mg/mL. To record the ^{1}H NMR, 0.30 mL of this solution was further diluted with 0.30 mL of DMSO-d_6.

HRMS experiments

HRMS spectra were recorded at the University of Glasgow on a Bruker micrOTOFQ instrument. For HRMS, initially gels were prepared using DMSO-d_6 and D_2O following the same methodology as described before. Then certain amounts of the gels were dissolved in DMSO-d_6 in such a way that the concentration of the gels became 318 mg/mL. To record the the mass spectra, 0.05 mL of this solution was further diluted with 0.5 mL of CH_3CN.

UV-Vis measurements

Absorption spectra of **1** and **2** under different conditions were recorded on Agilent Technologies Cary 60 UV-Vis spectrophotometer using a 0.01 mm path length quartz cuvette. All the gel samples were prepared in respected volumes in Sterilin vials using the same methodology as described before and were immidiately transferred to the cuvette for measurement.

Fluorescence spectroscopy

Emission spectra of **1** and **2** under different conditions were recorded on Agilent Technologies fluorescence spectrofluorophotometer. Samples were prepared in a PMMA cuvette with a path length of 1 cm by following the same procedure as mentioned before. For **2**, the excitation wavelength was 300 nm. For rest of the cases, the excitation wavelength was 350 nm. In all cases, both the excitation and emission slit widths were 20 nm.

Supplementary Figures

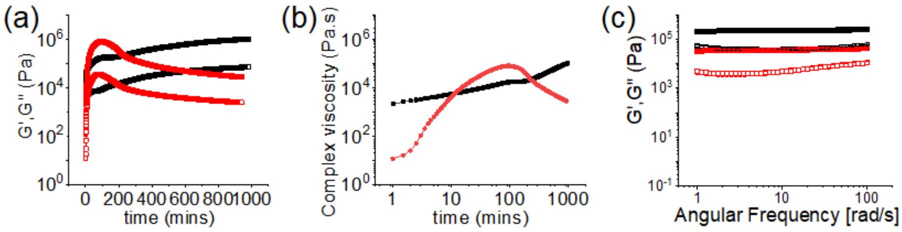

Figure S1. (a) Variation of G′ (closed symbol) and G″ (open symbol) with time for the mixture of **1** and **2** in absence (black) and presence (red) of Fe(II) (with the x-axis on a linear scale). Figure S1a represents Figure 2b of the manuscript using a linear X-scale. (b) Variation of complex viscosity with time for the mixture of **1** and **2** in absence (black) and presence (red) of Fe(II). (c) Frequency sweeps of the hydrogels obtained from the mixture of mixture of **1** and **2** in absence (black) and presence (red) of Fe(II). The closed symbols represent G′ and the open symbols represent G″. For (a-c), initial concentrations of **1**, **2** and Fe(II) are 0.134 M. Solvent is DMSO/H_2O (25/75, v/v). The frequency sweeps were collected after 16 hours.

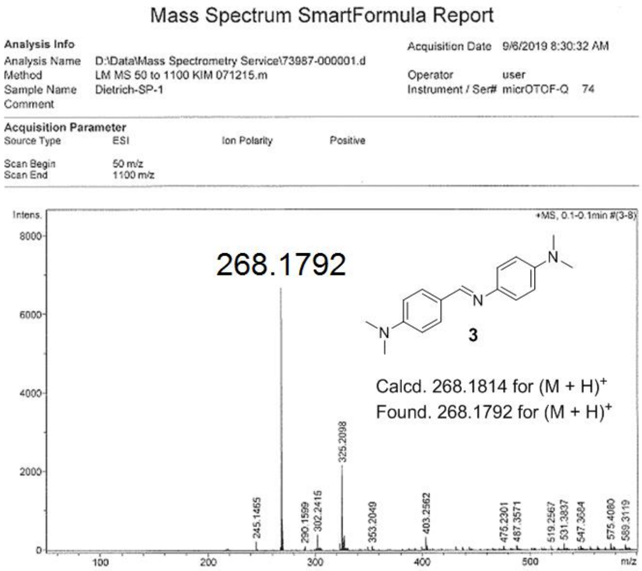

Figure S2. HRMS of the gel prepared from **1** and **2** (0.134 M).

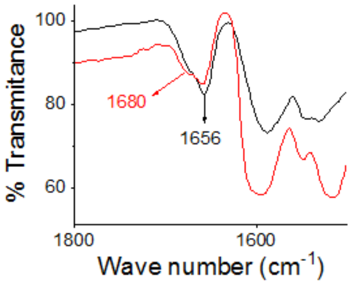

Figure S3. FTIR spectra of **1** (black) and the mixture of **1** and **2** (red). Initial concentrations of **1** and **2** are 0.134 M. Solvent is DMSO/H_2O (25/75, v/v).

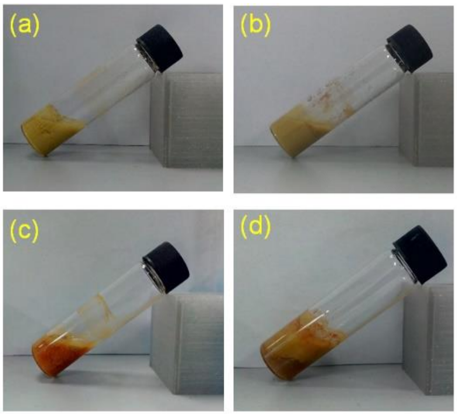

Figure S4. Photographs of the sols obtained from **3** in absence (a, b) and presence of (c, d) 1 equimolar amounts of Fe(II). Concentrations of **3** is 0.022 M (a, c) and 0.074 M (b, d). Solvent is DMSO/H_2O (25/75, v/v).

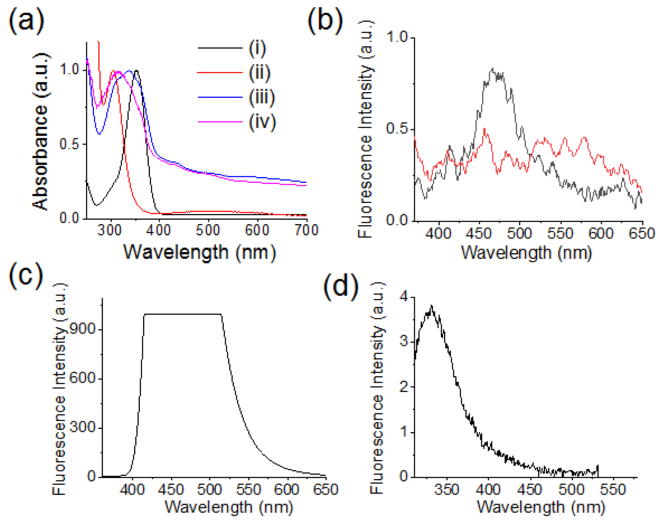

Figure S5. Normalized UV-vis spectra of sols of (i) **1**; (ii) **2** and the gels obtained from the mixture of **1** and **2** in absence (iii) and presence (iv) of Fe(II). (b) Comparison of emission spectra of the gels obtained from the mixture of **1** and **2** in absence (black) and presence (red) of Fe(II). (c) and (d) represent emission spectra of the sols of **1** and **2**. For (b) and (c), the excitation wavelength was 350 nm. For (d), the excitation wavelength used was 300 nm. For (a)-(d), the initial concentrations of **1**, **2** and Fe(II) are 0.134 M. Solvent is DMSO/H_2O (25/75, v/v).

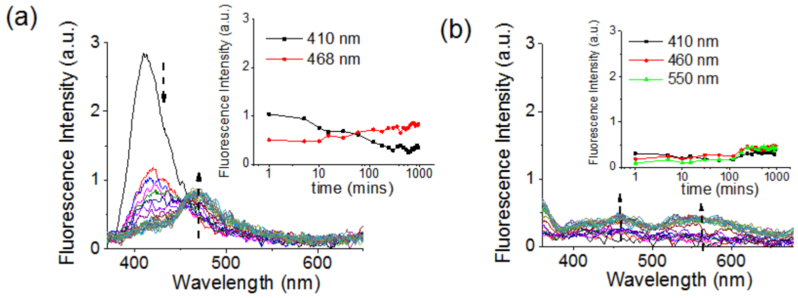

Figure S6. Change in emission with time for the hydrogels prepared from **1** and **2** in absence (a) and presence (b) of Fe(II). Insets represent change in emission intensity with time at respective wavelengths. In both cases, initial concentrations of **1**, **2** and Fe(II) are 0.134 M. Solvent is DMSO/H_2O (25/75, v/v).

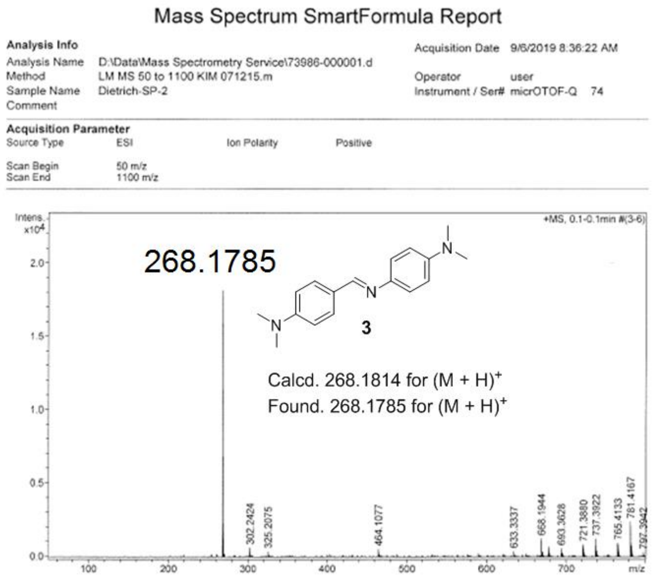

Figure S7. HRMS of the gel prepared from **1** and **2** in presence of Fe(II). Initial concentrations of **1**, **2** and Fe(II) are 0.134 M.

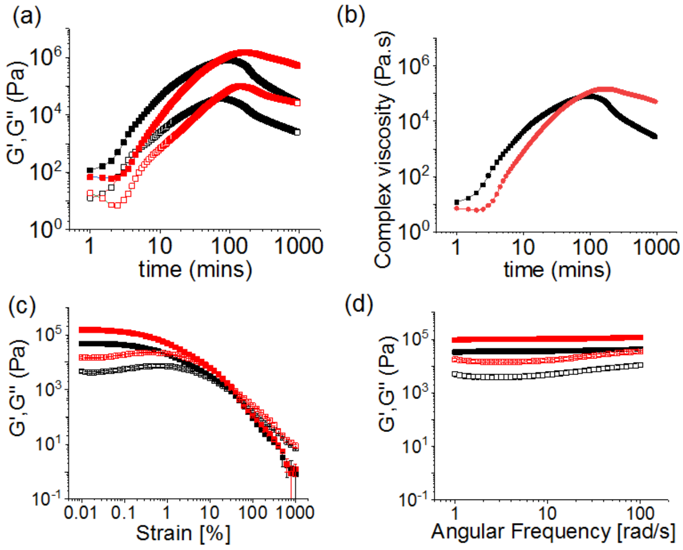

Figure S8. (a) Variation of G′ (closed symbol) and G″ (open symbol) with time for the mixture of **1** and **2** in presence of Fe(II). (b) Variation complex viscosity with time for the mixture of **1** and **2** in presence of Fe(II). (c) Strain sweeps and (d) frequency sweeps of the gels prepared in presence of Fe(II). The closed symbols represent G′, open symbols G″. For (a)-(d), In all cases, initial concentrations of **1**, **2** and Fe(II) are 0.134 M. Solvent is DMSO/H_2O (25/75, v/v). The black data is for normal water and the red data is for deoxygenated water.

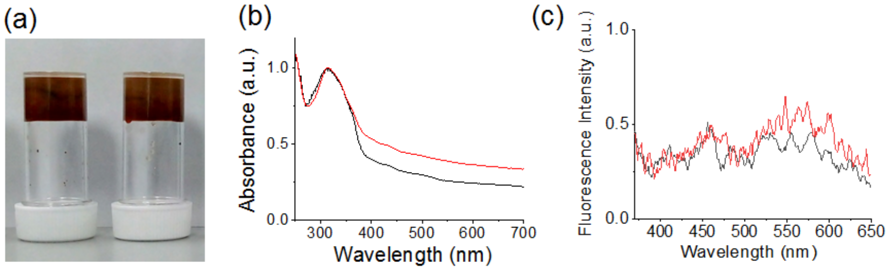

Figure S9: (a) Photograph of the gels obtained from the mixture of **1**, **2** and Fe(II) in presence (left) and absence (right) of dissolved oxygen. (b) Normalized UV-vis and (c) emission spectra of the gels prepared from **1** and **2** in presence of Fe(II). The black data is for normal water and the red data is for deoxygenated water. In all cases, initial concentrations of **1**, **2** and Fe(II) are 0.134 M. Solvent is DMSO/H_2O (25/75, v/v).

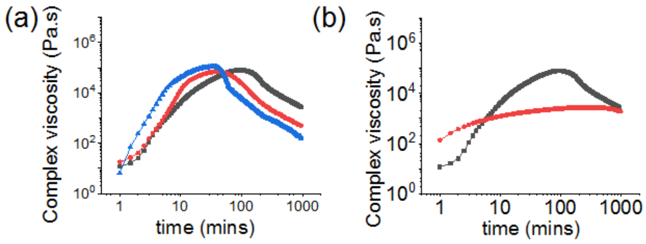

Figure S10. Variation of complex viscosity with time for the mixture of **1**, **2** and Fe(II) in presence of redox reaction involving (a) $NaNO_2$ and (b) H_2O_2. The black data is for no oxidising agent, the red data for 0.067 M and the blue data for 0.134 M oxidising agent. In all cases, initial concentrations of **1**, **2** and Fe(II) are 0.134 M. Solvent is DMSO/H_2O (25/75, v/v).

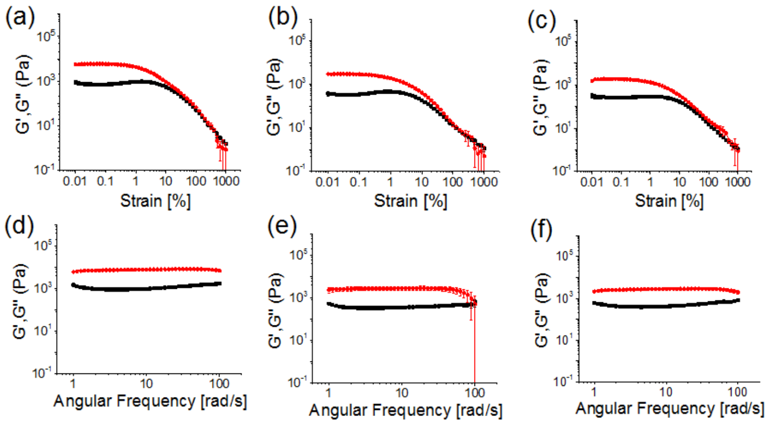

Figure S11. Strain sweeps (a-c) and frequency sweeps (d-f) of the gels obtained from the mixture of **1**, **2** and Fe(II) upon oxidation with 0.067 M of $NaNO_2$ (a, d), 0.067 M of H_2O_2 (b, e) and 0.134 M of $NaNO_2$ (c, f). In all cases, the red data represent G′, black data G″. Initial concentrations of **1**, **2** and Fe(II) are 0.134 M. Solvent is DMSO/H_2O (25/75, v/v).

Table S1. Comparison of frequency sweep data of gels of **1** prepared by different methods

Gel conditions		G′ (Pa) [at 10 rad/s]	Critical strain (%)	%Strain at crossover point
**1** alone		230000	0.2	100
Gel + Fe(II)	No dissolved oxygen	102165	0.25	20
	Normal water	36235	0.25	35
Gel + Fe(II) + 0.067 M of $NaNO_2$		8085	0.80	410
Gel + Fe(II) + 0.134 M of $NaNO_2$		2830	0.90	900
Gel + Fe(II) + 0.067 M of H_2O_2		2923	0.80	415

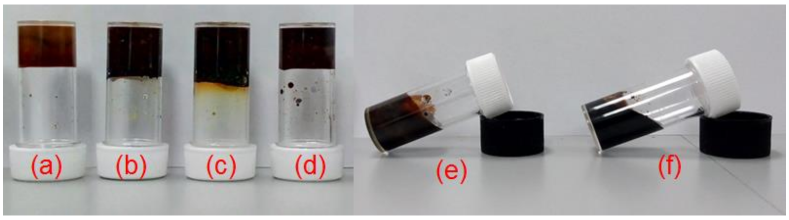

Figure S12. Photographs of the gels obtained from **1**, **2** and Fe(II) by redox reaction involving (a) no oxidising agent, (b) 0.067 M of $NaNO_2$, (c) 0.134 M of $NaNO_2$, (d) 0.067 M of H_2O_2. Photographs of the sols obtained from **1** and **2** in presence of (e) Fe(II) and 0.134 M of H_2O_2 and (f) Fe(III). In all cases, initial concentrations of **1**, **2**, Fe(II) and Fe(III) are 0.134 M. Solvent is DMSO/H_2O (25/75, v/v).

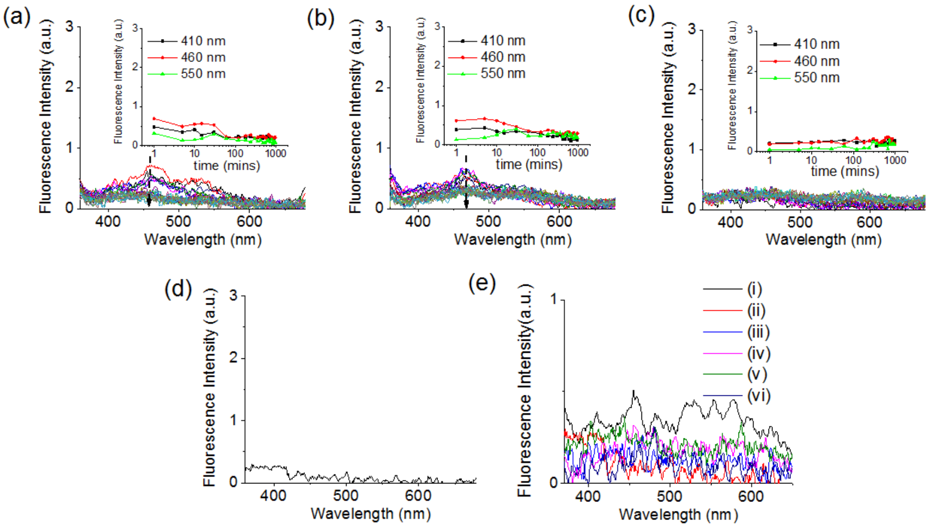

Figure S13. Change in emission with time for the for the hydrogels prepared from **1** and **2** in presence of Fe(II) involving oxidation reaction with 0.067 M of $NaNO_2$ (a), 0.134 M of $NaNO_2$ (b), and 0.067 M of H_2O_2 (c). Insets represent change in emission intensity with time at respective wavelengths. (d) Emission spectra of the sol obtained from the mixture of **1**, **2** and Fe(III). (e) Comparison of emission spectra of the gel (i) and sol (ii) obtained from the mixture of **1** and **2** in presence of Fe(II) and Fe(III) respectively. (iii)-(v) Represent emission spectra of the gels obtained from the mixture of **1**, **2** and Fe(II) in presence of redox reaction involving 0.067 M of $NaNO_2$ (iii), 0.134 M of $NaNO_2$ (iv) and 0.067 M of H_2O_2 (v). (vi) Represents Emission spectra of the sol obtained from the mixture of **1**, **2** and Fe(II) in presence of 0.134 M of H_2O_2. In all cases, initial concentrations of **1**, **2,** Fe(II) and Fe(III) are 0.134 M. Solvent is DMSO/H_2O (25/75, v/v).

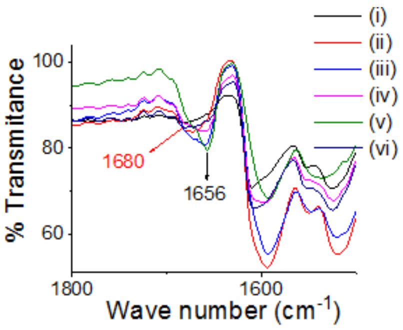

Figure S14. (i) FTIR spectrum of the gel from **1** and **2** in presence of Fe(II). (ii)-(iv) Represent the FTIR spectra of the gels obtained from the oxidation of Fe(II) by 0.067 M of $NaNO_2$ (ii), 0.134 M of $NaNO_2$ (iii) and 0.067 M of H_2O_2 (iv). (v) FTIR spectrum of the sol obtained from the oxidation of Fe(II) by 0.134 M of H_2O_2. (vi) FTIR spectrum of the sol obtained from the mixtures of **1**, **2** and Fe(III). In all cases, initial concentrations of **1**, **2,** Fe(II) and Fe(III) are 0.134 M. Solvent is DMSO/H_2O (25/75, v/v).

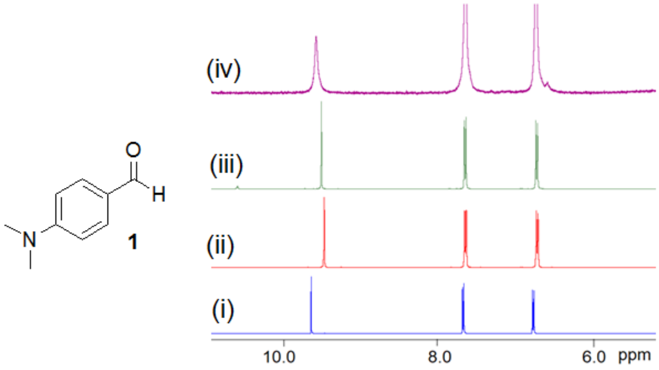

Figure S15. Partial ^{1}H NMR (in DMSO-d_6) spectra of (i) **1**, (ii) **1** with $NaNO_2$, (iii) **1** with H_2O_2 and (iv) mixture of **1** and Fe(II) with H_2O_2. We were unable to record the proton NMR of the mixture of **1** and Fe(II) with $NaNO_2$. Proton NMRs were recorded after 6h of addition of the oxidising agents. The peak at 10.58 ppm in (iii) is corresponds to H_2O_2. Initial concentrations of **1**, Fe(II), $NaNO_2$ and H_2O_2 are 0.134 M.

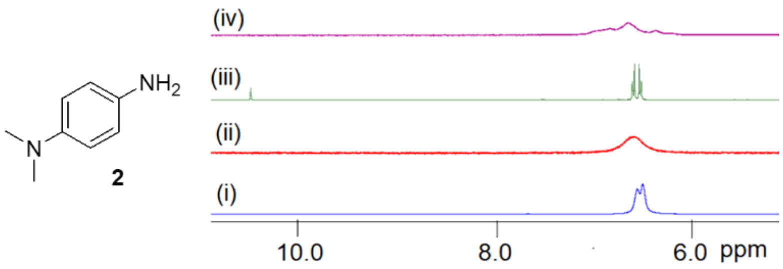

Figure S16. Partial ^{1}H NMR (in DMSO-d_6) spectra of (i) **2**, (ii) **2** with $NaNO_2$, (iii) **2** with H_2O_2 and (iv) mixture of **2** and Fe(II) with H_2O_2. We were unable to record the proton NMR of the mixture of **2** and Fe(II) with $NaNO_2$. Proton NMRs were recorded after 6h of addition of the oxidising agents. The peak at 10.48 ppm in (iii) is corresponds to H_2O_2 which disappeared in (iv) due to oxidation of Fe(II). Initial concentrations of **2**, Fe(II), $NaNO_2$ and H_2O_2 are 0.134 M.

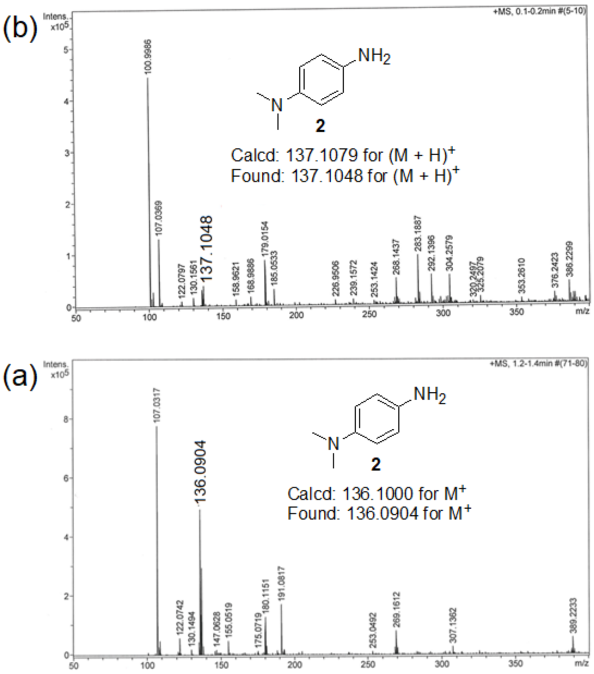

Figure S17. HRMS of the sols prepared from (a) **2** with H_2O_2 and (b) **2** and Fe(II) with H_2O_2. Initial concentrations of **2**, Fe(II) and H_2O_2 are 0.134 M.

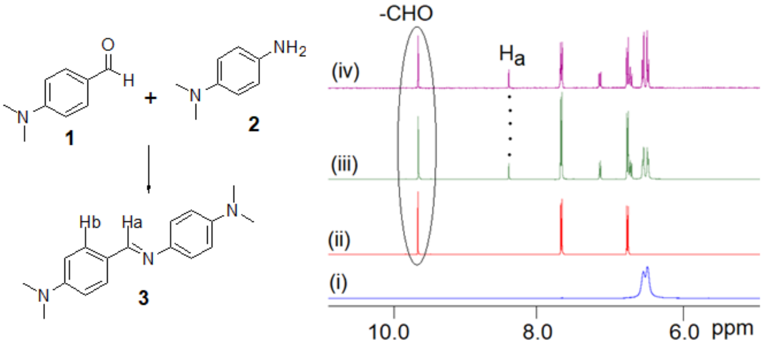

Figure S18. Partial ^{1}H NMR (in DMSO-d_6) spectra of (i) **2**, (ii) **1**, (iii) gel obtained from the mixture of **1** and **2** in absence of $NaNO_2$, (iv) gel obtained from the mixture of **1** and **2** in presence of $NaNO_2$. Initial concentrations of **1**, **2** and $NaNO_2$ are 0.134 M.

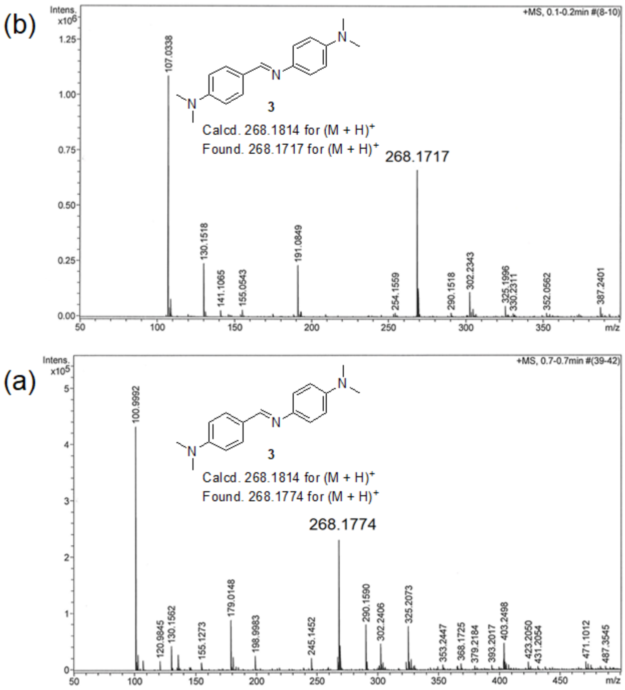

Figure S19. HRMS of the gels prepared from the mixture of **1**, **2** and $NaNO_2$ in absence (a) and presence (b) of Fe(II). Initial concentrations of **1**, **2**, Fe(II) and $NaNO_2$ are 0.134 M.

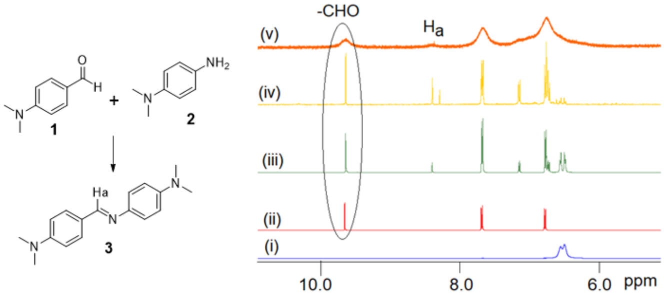

Figure S20. Partial ^{1}H NMR (in DMSO-d_6) spectra of (i) **2**, (ii) **1**, (iii) gel obtained from the mixture of **1** and **2** in absence of H_2O_2, (iv) gel obtained from the mixture of **1** and **2** in presence of H_2O_2, (v) sol obtained from the mixture of **1**, **2** and Fe(II) in presence of H_2O_2. In all cases, the initial concentrations of **1**, **2**, Fe(II) and H_2O_2 are 0.134 M.

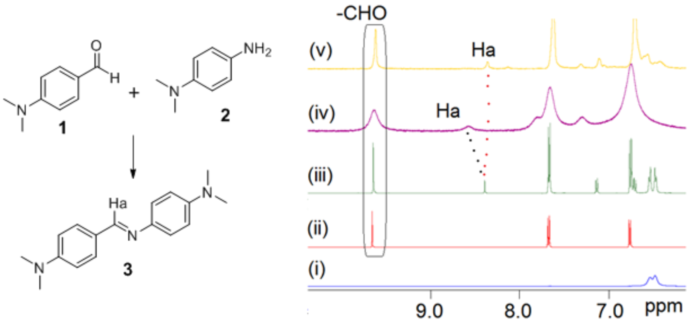

Figure S21. Partial ^{1}H NMR (in DMSO-d_6) of **2** (i), **1** (ii), gels obtained from the mixture of **1** and **2** in absence (iii) and presence (iv) of Fe(II), and the sol obtained from the mixture of **1** and **2** in presence of Fe(III) (iv). In all cases, initial concentrations of **1**, **2,** Fe(II) and Fe(III) are 0.134 M.

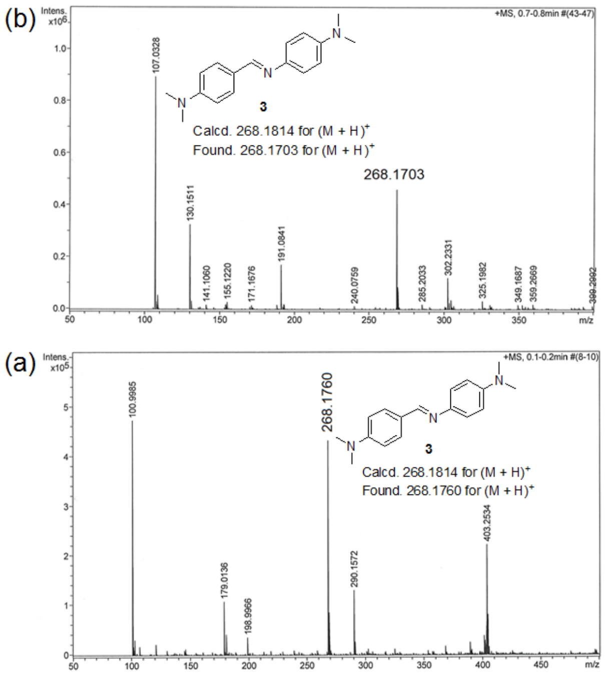

Figure S22. HRMS of the gel and sol prepared from from the mixture of **1**, **2** and H_2O_2 in absence (a) and presence (b) of Fe(II) respectively. Initial concentrations of **1**, **2** and $NaNO_2$ are 0.134 M.

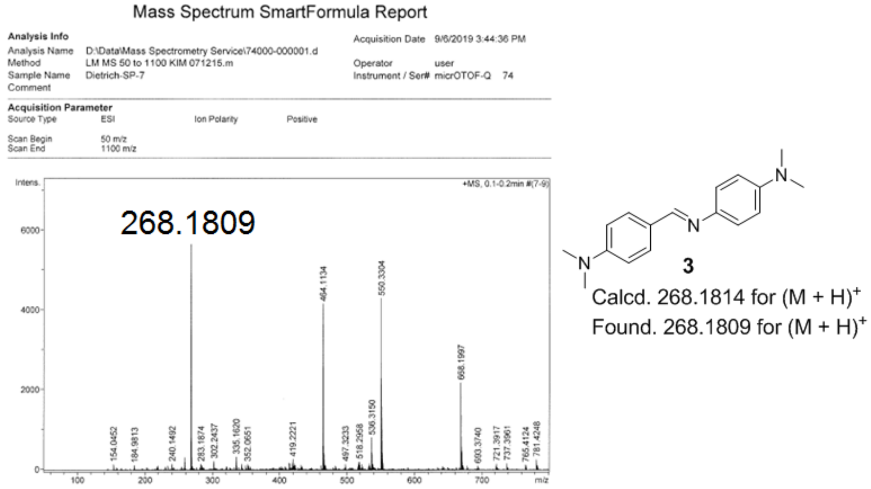

Figure S23. HRMS of the sol prepared from **1**, **2** and Fe(III) (0.134 M).

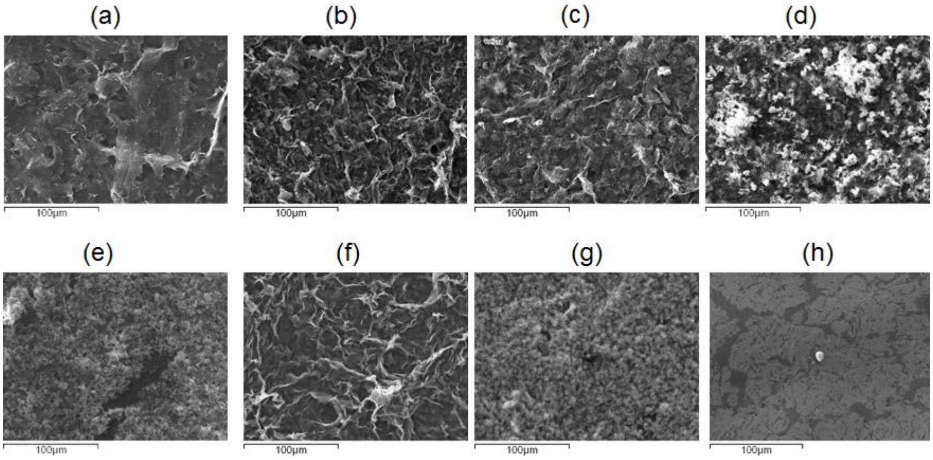

Figure S24. SEM images of the xerogels (a-f) and sols (g, h) obtained from **1** and **2** under different conditions: (a) xerogel from **1** and **2**. (b) and (c) Xerogels from **1** and **2** in presence of Fe(II) with normal water (b) and water with no dissolve oxygens (c). (d)-(f) Xerogels obtained from the oxidation of Fe(II) involving 0.067 M of $NaNO_2$ (d), 0.134 M of $NaNO_2$ (e) and 0.067 M of H_2O_2 (f). (g) Sol obtained from the oxidation of Fe(II) by 0.134 M of H_2O_2. (g) Sol obtained from the mixtures of **1**, **2** and Fe(III). In all cases, initial concentrations of **1**, **2,** Fe(II) and Fe(III) are 0.134 M. Solvent is DMSO/H_2O (25/75, v/v).

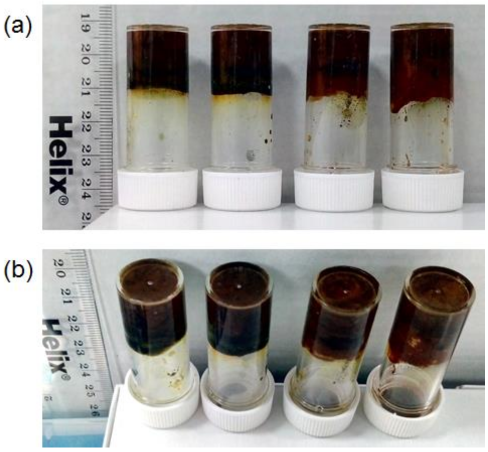

Figure S25. Photographs showing the ability of the gels towards swelling before being unable to withstand the expansion. Gels are prepared from **1, 2** in presence of Fe(II) involving oxidation reaction by $NaNO_2$ [(a) front view, (b) top view]. In each picture, from left to right, initial concentration of NaNO2 is 0.134 M, 0.268 M, 0.402 M and 0.670 M. In all cases, initial concentrations of **1**, **2** and Fe(II) are 0.134 M. Solvent is DMSO/H_2O (25/75, v/v).
